# Physical fitness characteristics and neck and shoulder pain incidence in school‐aged children—A 2‐year follow‐up

**DOI:** 10.1002/hsr2.852

**Published:** 2022-10-08

**Authors:** Katariina Pauliina Pirnes, Jouni Juhani Kallio, Harto Juho Hakonen, Arto Jorma Hautala, Laura Joensuu, Arja Helena Häkkinen, Tuija Heini Tammelin

**Affiliations:** ^1^ Faculty of Sport and Health Sciences University of Jyväskylä Jyväskylä Finland; ^2^ Likes, School of Health and Social Studies Jamk University of Applied Sciences Jyväskylä Finland

**Keywords:** neck and shoulder pain incidence, physical fitness characteristics, school‐aged children

## Abstract

**Background and Aims:**

Neck and shoulder pain (NSP) is common in school age, but preventative factors have not been identified. The purpose was to study whether a fitness test could be used to predict the incidence of NSP and determine whether good physical fitness characters would be associated with lower NSP incidence in school‐aged children at 2‐year follow‐up.

**Methods:**

After the invitation to nine schools, 970 children (10–15 years old) agreed to participate. Flexibility, fundamental movement skills, musculoskeletal fitness, and cardiorespiratory fitness measurements included in Finnish Schools on the Move! monitoring system for physical functional capacity were measured at baseline in 2013. The NSP incidence was assessed by an online survey during school hours after 1 and 2 years. Logistic regression was used to analyze associations between physical fitness characteristics and NSP incidence.

**Results:**

The mean prevalence of NSP was 26% at baseline. The NSP incidence was 15% in the first and 18% in the second follow‐up year. Good physical fitness was not associated with lower NSP incidence in the 2‐year follow‐up. Successful lower back extension (odds ratio [OR] = 2.83) and good scores in curl‐up (OR = 1.80) adjusted with age, gender, and body mass index, were associated with higher NSP incidence between T0 and T2. Throwing–catching combination (OR = 0.55) was associated with a lower NSP incidence in unadjusted analysis, but the association did not remain after adjustments.

**Conclusion:**

Good physical fitness characteristics were not consistently associated with a lower NSP incidence in school‐aged children in a 2‐year follow‐up. The role of general field‐based physical fitness test as a screening tool for NSP incidence remains unconfirmed. More longitudinal studies are needed to detect the factors underlying NSP incidence in school‐aged children.

## 1 INTRODUCTION

Despite the global improvement of the overall health between 1990 and 2019, musculoskeletal disorders in general have an ascending trend.^1^ Nevertheless, neck and shoulder pain (NSP) in school‐aged children has become a serious, growing problem that might lead to potential health‐related problems at early adulthood.^2^ NSP have been found to be persistent in nature^3^ and some evidence suggests that adolescents with neck symptoms are at higher risk of developing NSP in adulthood.^4^ However, the prognostic factors behind the increasing incidence of NSP have not been adequately studied in school‐aged children. Since general physical fitness measurements are widely used in schools, the usefulness of the information obtained from them in health education and health monitoring should be investigated in front of growing health issues that NSP now is.

There are no longitudinal studies on the role of childhood/adolescent physical fitness on the incidence of NSP. Longitudinal studies in adults have not found clear associations between physical fitness characteristics and NSP, while associations between physical fitness and other factors such as social support or work‐related factors have been found.^5^, ^6^, ^7^ Some longitudinal studies have reported interesting associations between physical fitness and NSP incidence from childhood to adulthood.^8^, ^9^ The results of a 25‐year study on predictive values of measured fitness characteristics to adult pain conditions showed, that boys in the best tertile of flexibility had the least tension neck symptoms as adults, while girls in the best flexibility tertile were most likely to have tension neck symptoms as adults.^9^ Additionally, good performance in bench press at the age of 16 has been reported to be associated with a lower risk for neck–shoulder symptoms among men.^8^

Musculoskeletal health is, for example, according to the Toronto model, one factor closely related to health‐related fitness^10^ and therefore the interactions should be carefully studied. Plowman et al. (2014) have emphasized the importance of longitudinal physical fitness studies performed only in children and adolescents to find associations between potential health risk factors and valid, reliable field tests.^11^ This is justified because the philosophy of physical fitness testing for adolescents has changed from performance‐based to health‐based assessment a few decades ago.^12^ Current studies in children and adolescents are mainly in cross‐sectional settings, but based on them, good physical characteristics might be negatively associated with NSP.^13^, ^14^, ^15^

Physical fitness measurements should be able to measure the range from limited function to high capacity and accurately reflect the characteristics of the child's physical fitness.^12^ Measurements should also take into account the possible effects of modifying factors such as age, sex, and body composition on the associations studied.^12^ All these factors have been considered in the present study design. Because the effects of fitness characteristics on the incidence of NSP in school‐aged children are not well known, we tested a hypothesis in which good physical fitness would be associated with a lower incidence of NSP in school‐aged children during the 2‐year follow‐up. To assess this, we utilized the field‐based fitness measurements included in the Finnish national Move! monitoring system for physical functional capacity aimed for school‐aged children.

## 2 MATERIALS AND METHODS

### 2.1 Participants

This study is part of the larger research project linked to national “Finnish Schools on the Move” program^16^, ^17^, ^18^, ^19^, ^20^ and a total of 1710 school‐aged children in grades 4–7 from nine public schools across Finland were invited to participate in a longitudinal study (2013–2015). Of these children, 970 participated (mean 12.5 years ± 1.3 years; 52.5% girls) and 684 (75.6%) provided information on all the study variables at baseline. After excluding children who reported NSP due to spinal injuries, the final sample of this cohort comprised 905 10–15‐year‐old children. In accordance with the Declaration of Helsinki, a written informed consent was obtained from all the children and their guardians before participation in the study. The study setting was approved by the Ethics committee of the University of Jyväskylä (January 2012).

### 2.2 Measurements

The participants filled in a web‐based questionnaire in spring 2013, 2014, and 2015. The test–retest repeatability of the NSP questionnaire has been reported to be substantial (Kappa [κ] value 0.68 for the 2‐point scale and intraclass correlation coefficient [ICC] 0.67 for the 5‐point scale).^19^ Data on children's NSP were collected with a question that illustrates pain in the last 3 months before the study: “How often have you had symptoms in the last three months”? The children chose the appropriate frequency and selected areas of the body, such as “neck or shoulder pain or ache,” from the list. The answer options were selected from five categories: (1) almost daily, (2) more than once a week, (3) about once a week, (4) once a month, and (5) rare or never. The questionnaire included a figure of the human body with zones and written names of the corresponding body areas to ensure that the different body regions were understood correctly. Participants were allowed to ask for help in completing the questionnaire from an adult in the class.

For the analysis of the incidence, the answers regarding NSP symptoms during last 3 months were grouped into two categories: (1) once a week or more often and (2) less than once a week. The pupils were also asked if they had had pain originated from a trauma. Children who reported trauma in the neck and shoulder area (*n* = 42) were excluded in the analysis. The incidence of NSP in this study refers to new cases, where pain was reported as occuring at least once a week during the past 3 months.

Measurements of physical fitness characteristics at baseline were included. These measurements are part of the national Move! monitoring system for a physical functional capacity which is implemented in the physical education curriculum of Finnish fifth and eighth‐grade children.^21^ The main purpose of the use of this instrument in schools is to encourage children to independently take care of their physical functional capacity. The measured physical fitness characteristics are flexibility, muscular fitness, fundamental movement skills, and cardiorespiratory fitness.

*Flexibility* indicates the ability to move freely throughout the range of motion of joints.^11^ Flexibility^21^ was measured by four tasks consisting of multijoint flexibility measurements; lower back extension in sitting posture, squat, and right and left shoulder stretch. The tasks were evaluated according to the selected criteria (0= did not succeed, 1 = succeeded).

*Muscular fitness* reflects the ability to work against resistance^22^ and it was assessed with push‐up and curl‐up measurements. Push‐up^23^, ^24^ measures upper body strength. Boys performed push‐ups with hands and toes and girls with hands and knees on the ground. The children repeated the movement as many times as possible in 1 min time and only correctly performed repetitions were recorded. Curl‐up^25^, ^26^ is a modified version of the FitnessGram curl‐up and the number of correct repetitions was calculated with a maximum of 75 repetitions.

*Fundamental movements skills* (FMS) representing neuromotor skills include balance, coordination, gait, agility, and proprioceptive skills^27^ and were assessed with five‐leap and throwing–catching combination tests. In the five‐leap test,^25^ children tried to jump as far as possible with five consecutive jumps. The first leap was strained with both feet, followed by four alternating one‐foot leaps forward and the last leap ending on both feet. Best performance out of two attempts was recorded in meters to the nearest 0.1 m.

In the throwing–catching combination test,^21^ a tennis ball was thrown from 7 to 10 m distance (a distance selected for age and sex) to a 1.5 m × 1.5 m target area on the wall, 0.9 m above the floor. A successful throw‐catch combination included hitting the target area behind the marked line and grabbing the ball after one bounce. The number of successful throwing–catching combinations was counted.

*Cardiorespiratory fitness* represents the total capacity of the cardiovascular and respiratory organs and enables to carry out physically demanding tasks for a prolonged period of time.^22^ Cardiorespiratory fitness was estimated with the performance of the 20‐m shuttle run test where running speed is increased in 1‐min interval until maximal voluntary exhaustion. Initial speed was 8.0 km h^−1^, following speed 9.0 km h^−1^, and following increment of 0.5 km h^−1^ per stage.^28^ The result was counted as the number of laps run.

Measurements were performed by group of four to six educated research personnel, in the children's own school sports facilities. One group at a time (average 25 children) participated in the measurements during 1.5 h. Measurement techniques were explained to the participants and practiced before the formal evaluation. The test scores were recorded by the research staff.^16^

Each pupil's weight and height were measured and used to calculate the body mass index (BMI, kg ∙ m^−2^). Weight was measured in light clothing using bioelectrical impedance analysis (InBody 720, Biospace Co., Ltd). For measuring the height, a portable Charder HM 200 P measuring instrument was used. The measurement was done twice. If the results between the measurements differed by more than 0.4 cm, a third measurement was made. The average of the two closest results was used in the analysis.

### 2.3 Data analysis

Move! results at baseline (T0) and one‐year measurement point (T1) and the self‐reported weekly prevalence and incidence of NSP at three measurement points (T0, T1, and T2) were analyzed. The incidence of NSP was observed between T0 and T2, but also between T0–T1 and T1–T2. The data are presented as means, standard deviations (SD), and percentages.

The incidence of NSP refers to new cases, where pain was perceived at least once a week during past 3 months. The variable of NSP incidence received a value 0 if the participant still experienced NSP less than once a week (reference category) in the 1‐year follow‐up. If more frequent NSP were observed compared with the reference category, NSP incidence variable received a value 1. Children with NSP at least once a week already at baseline were not included in the analysis.

The unequal probabilities of selection (by age and gender) were taken into account by using sampling weights in the modeling. Using information on population structure obtained from Official Statistics of Finland,^29^ sampling weights were constructed. Parameters of the models were estimated by using full information maximum likelihood method (FIML)^30^ with robust standard errors (MLR). Missing data were assumed to be missing at random (MAR). Because data were clustered within schools and ages, standard errors were calculated by using special feature of Mplus (TYPE = COMPLEX).

For logistic regression analysis, Move! results in different physical fitness characteristics were classified as good (Tertile 3), followed by moderate (Tertile 2) and low (Tertile 1) in scores. Tertiles were as uniform in size as possible and were standardized by age and gender to prevent weighted result. The results were analyzed without adjustments (Model 1) and with age, gender, and BMI adjustments (Model 2) and the comparison was performed against to the Tertile 1. In the analysis of the flexibility measurements, tertiles were not needed since the variable was already dichotomous (did not succeed/succeeded) and the comparison was performed against a failed performance.

The dropouts were taken into account by using the FIML^30^ in the analyses, which means the model was corrected with a missing value. The analyzes were made by using SPSS 25.0 for Windows (SPSS Inc.) and Mplus 7.0 using a 5% significance level. *p* values ≤ 0.05 indicated a significant association.

## 3 RESULTS

### 3.1 Description of study population

Table 1 shows the characteristics of the participants and the differences between boys and girls. The mean prevalence of NSP at least once a week was 26% among all children at baseline. NSP incidence was an average of 15% between T0 and T1 and 18% between T1 and T2. There was a significant difference in the incidence of NSP between boys and girls between T0 and T1 (*p* = 0.006) but not between T1 and T2 (*p* = 0.207). All the physical fitness characteristics together differed between boys and girls (*p* = 0.008–<0.001), although in the push‐up for boys and girls and in the throwing–catching combination test for boys and girls and for different age groups was used a different adjustment in starting position.

**Table 1:** The characteristics for all the participants, boys, and girls and the differences between boys and girls

		TOTAL		BOYS		GIRLS		*p*
	Grade	*N*	Mean (sd)	*N*	Mean (sd)	*N*	Mean (sd)	(boys/girls)
Age (y)		970	12.5 (1.3)	462	12.6 (1.3)	507	12.5 (1.3)	0.605
BMI (kg ∙ m^2^)		914	18.9 (3.2)	429	18.6 (3.3)	485	19.1 (3.2)	0.054
NSP total		905	2.0 (1.1)	430	1.9 (1.0)	475	2.0 (1.1)	0.110
NSP (at least once a week)		905	26%	430	23%	475	28%	0.091
NSP incidence T0–T1		605	15%	294	11%	311	19%	0.006
NSP incidence T1–T2		586	18%	295	16%	291	20%	0.207
Squat	4	207	89%	98	86%	109	92%	0.168
	5	157	94%	63	92%	94	95%	0.510
	6	164	92%	89	89%	75	96%	0.087
	7	382	92%	179	89%	203	94%	0.138
	TOT	910	91%	429	89%	481	94%	0.008
Lower back extension	4	207	72%	98	62%	109	82%	0.002
	5	157	80%	63	67%	94	88%	0.001
	6	164	79%	89	72%	75	87%	0.022
	7	382	81%	179	75%	203	87%	0.005
	TOT	910	79%	429	70%	481	86%	<0.001
Shoulder strech/Right	4	207	88%	98	95%	109	82%	0.003
	5	157	89%	63	92%	94	86%	0.256
	6	164	93%	89	92%	75	95%	0.518
	7	381	89%	179	92%	202	86%	0.091
	TOT	909	89%	429	93%	480	86%	0.003
Shoulder strech/Left	4	207	59%	98	53%	109	64%	0.103
	5	157	66%	63	60%	94	69%	0.254
	6	164	60%	89	49%	75	72%	0.003
	7	382	69%	179	54%	203	82%	<0.001
	TOT	910	64%	429	54%	481	74%	<0.001
Push‐up	4	197	16.6 (12.7)	96	13.1 (12.4)	101	19.9 (12.1)	<0.001
	5	155	19.5 (12.6)	62	13.9 (9.6)	93	23.2 (13.1)	<0.001
	6	158	19.3 (11.0)	83	17.0 (11.5)	75	21.7 (9.9)	0.007
	7	367	23.5 (13.5)	172	19.5 (11.5)	195	27.1 (14.2)	<0.001
	TOT	877	20.5 (13.0)	413	16.7 (11.7)	464	23.9 (13.2)	<0.001
Curl‐up	4	205	29.8 (19.0)	97	29.2 (19.2)	108	30.4 (18.9)	0.637
	5	157	41.3 (21.9)	63	41.6 (22.3)	94	41.2 (21.9)	0.910
	6	161	41.4 (20.8)	88	42.6 (20.8)	73	39.8 (20.7)	0.391
	7	373	37.2 (19.6)	177	42.8 (19.8)	196	32.1 (18.0)	<0.001
	TOT	896	37.0 (20.5)	425	39.5 (21.0)	471	34.7 (19.9)	<0.001
Throwing–catching combination	4	206	9.5 (4.8)	98	10.3 (4.9)	108	8.8 (4.6)	0.030
	5	156	12.1 (5.4)	63	13.4 (5.4)	93	11.2 (5.2)	0.009
	6	164	14.1 (4.5)	89	14.6 (4.8)	75	13.5 (4.1)	0.128
	7	375	12.4 (4.4)	177	12.6 (4.5)	198	12.3 (4.2)	0.495
	TOT	901	12.0 (4.9)	427	12.6 (5.0)	474	11.5 (4.8)	<0.001
5‐leap	4	204	7.4 (0.9)	97	7.5 (0.9)	107	7.4 (0.9)	0.463
	5	154	7.9 (1.0)	63	8.2 (0.9)	91	7.7 (1.0)	0.003
	6	157	8.2 (1.0)	83	8.4 (1.1)	74	8.1 (0.8)	0.089
	7	367	8.8 (1.1)	173	9.2 (1.1)	194	8.4 (0.9)	<0.001
	TOT	882	8.2 (1.1)	416	8.5 (1.2)	466	8.0 (1.0)	<0.001
20‐m shuttle run	4	206	34.7 (16.3)	98	37.6 (17.6)	108	32.1 (14.6)	0.015
	5	152	40.3 (17.6)	63	47.5 (18.4)	89	35.3 (15.1)	<0.001
	6	158	42.4 (19.8)	84	47.3 (22.3)	74	36.9 (14.7)	<0.001
	7	355	46.5 (19.1)	165	53.4 (19.4)	190	40.6 (16.7)	<0.001
	TOT	871	41.9 (18.9)	410	47.5 (20.3)	461	37.0 (15.9)	<0.001

*Note*: NSP total, range 1–5*: mean answer: 1, rare or never; 2, about once a month; 3, once a week; 4, more than once a week; 5, almost daily.

Abbreviations: BMI, body mass index; NSP, neck and shoulder pain.

### 3.2 Associations of flexibility with the NSP incidence

Table 2 presents the associations between the dichotomous (did not succeed/succeeded) flexibility measurements and the incidence of NSP in unadjusted and adjusted (age, gender, and BMI) models at the follow‐up points. Successful lower back extension was associated with higher incidence of NSP (OR = 3.30) compared against the failed performances between T0 and T2. The association remained significant after adjustment (OR= 2.83). No other marked associations between the flexibility measurements and the NSP incidence were found.

**Table 2:** Logistic regression analysis between flexibility and neck and shoulder pain incidence in school‐aged children during 2‐year follow‐up

Incidence			Lower back extension			Squat			Shoulder strech/Right			Shoulder strech/Left		
			OR	95% CI	*p*	OR	95% CI	*p*	OR	95% CI	*p*	OR	95% CI	*p*
		No	1	1	‐	1	1	‐	1	1	‐	1	1	‐
T0–T2	Model 1	Yes	3.30	1.49–7.28	**0.003**	2.48	0.82–7.52	0.107	1.43	0.59–3.44	0.431	1.36	0.79–2.36	0.267
	Model 2	Yes	2.83	1.28–6.25	**0.010**	2.05	0.68–6.21	0.204	1.65	0.67–4.09	0.279	1.18	0.68–2.05	0.560
T0–T1	Model 1	Yes	1.56	0.66–3.68	0.308	0.72	0.34–1.51	0.384	1.43	0.58–3.51	0.436	0.95	0.53–1.70	0.864
	Model 2	Yes	1.30	0.58–2.90	0.524	0.55	0.27–1.13	0.103	1.63	0.70–3.80	0.254	0.83	0.48–1.43	0.499
T1–T2	Model 1	Yes	1.74	0.86–3.54	0.125	1.27	0.37–4.33	0.701	1.79	0.80–3.99	0.155	1.07	0.65–1.77	0.795
	Model 2	Yes	1.65	0.81–3.34	0.166	1.20	0.34–4.22	0.781	1.84	0.81–4.16	0.143	0.99	0.58–1.69	0.962

*Note*: Statistically significant results are bolded.

Abbreviations: Model 1, crude analysis; Model 2, adjusted with age, gender and BMI (body mass index); No, did not succeed; Yes, succeeded; T0, baseline; T1, first follow‐up year; T2, second follow‐up year.

### 3.3 Associations of muscular fitness, fundamental motor skills, and cardiorespiratory fitness with the NSP incidence

Table 3 shows the associations between muscular fitness, fundamental movement skills, and cardiorespiratory fitness and the NSP incidence at the follow‐up points. The Tertile 3 with good scores in curl‐up had an association with a higher risk of NSP incidence (OR = 1.75) in unadjusted model and when the model was adjusted, the Tertile 2 had an association with a higher risk of NSP incidence (OR = 1.80) compared with the Tertile 1 with low scores at T0–T2. The children in Tertile 3 in throwing–catching combination test, were less likely to get NSP (unadjusted OR = 0.55) compared with children in the Tertile 1 between T0 and T2. However, this association did not remain after adjustments. No other marked associations in follow‐up points between these physical fitness characteristics and the NSP incidence were found.

**Table 3:** Logistic regression analysis between muscular fitness, fundamental movement skills, cardiorespiratory fitness, and NSP incidence in school‐aged children during 2‐year follow‐up

Incidence			Push‐up			Curl‐up			Throwing–catching combination			5‐leap			20‐m shuttle‐run		
			OR	95% CI	*p*	OR	95% CI	*p*	OR	95% CI	*p*	OR	95% CI	*p*	OR	95% CI	*p*
		Tertile1	1	1	‐	1	1	‐	1	1	‐	1	1	‐	1	1	‐
T0‐T2	Model 1	Tertile2	1.33	0.76–2.32	0.320	1.19	0.66–2.16	0.569	1.06	0.65–1.73	0.822	1.13	0.66–1.91	0.662	1.56	0.83–2.94	0.171
		Tertile3	1.52	0.78–2.98	0.219	1.75	1.01–3.04	**0.048**	0.55	0.31–0.97	**0.038**	1.00	0.59–1.71	0.994	1.08	0.60–1.95	0.791
	Model 2	Tertile2	1.33	0.70–2.53	0.377	1.24	0.65–2.35	0.511	1.09	0.66–1.82	0.729	1.14	0.64–2.02	0.656	1.53	0.79–2.98	0.209
		Tertile3	1.60	0.79–3.24	0.190	1.80	1.02–3.16	**0.042**	0.59	0.34–1.03	0.065	1.05	0.61–1.82	0.853	1.11	0.60–2.07	0.736
T0‐T1	Model 1	Tertile2	1.37	0.75–2.50	0.300	0.73	0.36–1.45	0.365	1.13	0.63–2.02	0.675	0.84	0.44–1.60	0.600	1.47	0.74–2.89	0.269
		Tertile3	1.20	0.67–2.17	0.541	1.46	0.76–2.79	0.255	0.68	0.35–1.31	0.244	0.92	0.53–1.61	0.776	0.81	0.40–1.67	0.570
	Model 2	Tertile2	1.39	0.70–2.76	0.343	0.79	0.40–1.56	0.494	1.19	0.67–2.12	0.544	0.85	0.41–1.74	0.648	1.46	0.73–2.93	0.290
		Tertile3	1.25	0.66–2.36	0.492	1.47	0.77–2.83	0.246	0.75	0.39–1.42	0.372	0.97	0.53–1.77	0.925	0.76	0.36–1.63	0.484
T1‐T2	Model 1	Tertile2	1.43	0.77–2.67	0.258	1.31	0.745–2.31	0.341	0.97	0.57–1.68	0.925	0.79	0.48–1.30	0.350	1.28	0.68–2.41	0.444
		Tertile3	1.19	0.64–2.21	0.587	1.33	0.73–2.43	0.358	0.85	0.49–1.46	0.547	0.71	0.38–1.33	0.279	1.15	0.55–2.39	0.708
	Model 2	Tertile2	1.39	0.71–2.74	0.339	1.21	0.68–2.16	0.508	0.97	0.55–1.72	0.921	0.76	0.46–1.24	0.269	1.18	0.63–2.22	0.610
		Tertile3	1.09	0.57–2.10	0.788	1.20	0.63–2.29	0.573	0.89	0.52–1.53	0.676	0.68	0.36–1.29	0.239	1.06	0.50–2.23	0.884

*Note*: Statistically significant results are bolded.

Abbreviations: Model 1, crude analysis; Model 2, adjusted with age, gender and BMI (body mass index); T0, baseline; T1, first follow‐up year; T2, second follow‐up year; Tertile 1, low scores, a reference category; Tertile 2, moderate scores; Tertile 3, high scores.

## 4 DISCUSSION

NSP is a significant problem at school age, as evidenced by the 26% prevalence and the 15%–18% incidence during the 2‐year follow‐up presented in this study. We wanted to know whether good scores in a field‐based test for physical fitness characteristics would determine lower risk for the incidence of NSP in children. Contrary to our hypothesis, good scores on the physical fitness test did not predict a lower incidence of NSP pain among school‐aged children. Instead, successful lower back extension increased the likelihood of NSP by 2.8‐fold, and good scores in curl‐up increased the likelihood by 1.8‐fold. The participants who had good scores in throwing–catching combination test had a 45% lower risk of developing NSP, but the association was not remained after adjustments.

Detecting the associations to the incidence of NSP by measuring physical fitness characteristics can be skewed because the best measurement result does not always require good physical fitness. Someone in very good cardiorespiratory fitness, maybe not succeed in flexibility tasks and someone with poor cardiorespiratory fitness can perform these measurements easily. Such multidirectional associations between physical fitness characteristics and NSP has been reported, for example, Perry et al.^13^ in their cross‐sectional study for 1608 adolescents. Boys had higher odds for developing NSP when they threw basketball higher and jumped further^13^ and girls had higher odds for NSP if they had better abdominal endurance and two‐handed dexterity.^13^ In addition, the likelihood of pain increased with good back muscle endurance among girls but also with reduced back muscle endurance.^13^

The relationship of fitness characteristics to the incidence of NSP, especially in terms of flexibility, was reported by Mikkelsson et al. in a 25‐year follow‐up study where the young subjects were 16‐year‐old at baseline.^9^ Flexibility, assessed with sit and reach‐test, was found to be associated with a lower likelihood of tension neck symptoms in adulthood in men, while in women, good flexibility was associated with increased tension neck symptoms later in life.^9^ In our 1‐ and 2‐year follow‐up, flexibility measurements were performed differently than in Mikkelsson et al.^9^ study, where the sit and reach‐test were used. In addition, they also had a longer follow‐up. For these reasons, a more detailed comparison with their study is not justified.

The lower back extension in our study was performed sitting on the floor, legs straight together, and extending the lower back and the squat was performed similarly as an overhead squat by raising and holding arms straight up during the movement. In the shoulder stretch, one hand was reached down over the shoulder and reached behind the back upward with the other hand, trying to touch the fingers together. Although the evidence is not yet clear, flexibility has been used in health‐related fitness testing for children and adolescents since the 1980s.^31^ It is possible that flexibility may be related to a variety of health issues, such as back pain and injury prevention, but appropriate studies and data are still needed to establish such associations and, for example, to set cut‐off values for different flexibility measurements, as other fitness measurements already have.^31^

A previous cross‐sectional study in secondary school students suggested that health professionals should use physical fitness assessment as a tool to assess pain intensity.^32^ However, in light of current knowledge and our study results we do not support the suggestion of the usefulness of field‐based physical fitness testing as a tool to predict the incidence of NSP in school‐aged children. If children's NSP continues to grow, it may be necessary to develop a separate assessment tool for NSP that could be used in learning environments alongside the Move! or some other field‐based test. It seems, that measurements of general physical fitness do not appear to be sufficient in predicting NSP incidence in school‐aged children, but since physical fitness is often measured in schools, learning environments may play an important role in recognizing pain symptoms and promoting children toward an active lifestyle. Move! as a part of the national physical performance monitoring system in Finland, has been included in the school curriculum and designed specifically to support and encourage school‐aged children in terms of their physical functioning.^21^

### 4.1 Study strengths and limitations

The current study has many strengths such as the prospective setting, large sample size, and a well repeatable web‐based questionnaire as a self‐report tool.^19^ Associations have been studied broadly for different fitness characteristics and not only for one characteristic alone. To eliminate the effects of seasonal variation, the measurements were performed at the same time of year. However, a 2‐year follow‐up may be too long time to find stable associations with NSP incidence in school‐aged children, since NSP is fluctuating in nature^33^ and the recall time for the NSP was 3 months at the measurement points. The questionnaire may need to be repeated more frequently in future surveys to gain an understanding of, for example, the annual incidence of NSP.

## 5 CONCLUSIONS

Although the health benefits of good physical fitness are very clear for growing children, in the present study, good physical fitness characteristics were not associated with lower NSP incidence in 2‐year follow‐up. Therefore, the evidence does not support the use of field‐based fitness measurements as a screening tool for future NSP. As the prevalence and incidence of NSP are considerable high in this age‐group, we would like to suggest that in the onset of NSP, the etiology and treatment of the NSP nature would be determined individually for example using expertise offered by a physiotherapist. The inconsistent associations between some fitness characteristics and NSP incidence in this study encourage further research focusing on a broader search for factors underlying the NSP incidence to find preventive tools for school‐aged children for possible onset of NSP symptoms.

## AUTHOR CONTRIBUTIONS

**Katariina P. Pirnes**: Conceptualization; formal analysis; methodology; project administration; resources; validation; visualization; writing – original draft; writing – review & editing. **Jouni J. Kallio**: Conceptualization; supervision; validation. **Harto J. Hakonen**: Data curation; formal analysis; software. **Arto J. Hautala**: Conceptualization; supervision; writing – review & editing. **Laura Joensuu**: Conceptualization; supervision; validation; writing – review & editing. **Arja H. Häkkinen**: Conceptualization; supervision; validation; writing – review & editing. **Tuija H. Tammelin**: Conceptualization; data curation; formal analysis; funding acquisition; supervision; validation; writing – review & editing.

## CONFLICT OF INTEREST

The authors declare no conflict of interest.

## TRANSPARENCY STATEMENT

The correspondent author (Katariina P. Pirnes) of this study, affirms that this manuscript is an honest, accurate, and transparent account of the study being reported; that no important aspects of the study have been omitted; and that any discrepancies from the study as planned have been explained.

## Data Availability

The data that support the findings of this study are available from the corresponding author upon reasonable request.
